# EMT-Inducing Transcription Factors, Drivers of Melanoma Phenotype Switching, and Resistance to Treatment

**DOI:** 10.3390/cancers12082154

**Published:** 2020-08-04

**Authors:** Yaqi Tang, Simon Durand, Stéphane Dalle, Julie Caramel

**Affiliations:** 1 Cancer Cell Plasticity in Melanoma Laboratory, Université de Lyon, Université Claude Bernard Lyon 1, INSERM 1052, CNRS 5286, Centre Léon Bérard, Centre de Recherche en Cancérologie de Lyon, 69008 Lyon, France; yaqi.tang@lyon.unicancer.fr (Y.T.); simon.durand@lyon.unicancer.fr (S.D.); stephane.dalle@chu-lyon.fr (S.D.); 2 Dermatology Unit, Hospices Civils de Lyon, Centre Hospitalier Lyon Sud, 69495 Pierre Bénite, France

**Keywords:** EMT, transcription factors, melanocyte differentiation, melanoma development, phenotype switching, neural-crest stem cells, ZEB2/ZEB1 switch, intra-tumor heterogeneity, resistance to treatment

## Abstract

Transcription factors, extensively described for their role in epithelial–mesenchymal transition (EMT-TFs) in epithelial cells, also display essential functions in the melanocyte lineage. Recent evidence has shown specific expression patterns and functions of these EMT-TFs in neural crest-derived melanoma compared to carcinoma. Herein, we present an update of the specific roles of EMT-TFs in melanocyte differentiation and melanoma progression. As major regulators of phenotype switching between differentiated/proliferative and neural crest stem cell-like/invasive states, these factors appear as major drivers of intra-tumor heterogeneity and resistance to treatment in melanoma, which opens new avenues in terms of therapeutic targeting.

## 1. Introduction

The plasticity of tumors is a major source of intra-tumor heterogeneity, which underlies their capacity to adapt to the selective pressures they encounter at every stage of tumor development, from early stages of tumor initiation, through disease progression, and in response to therapy. The epithelial–mesenchymal transition (EMT) is an essential embryonic process, which provides motility properties and drives the reversible reprogramming of polarized epithelial cells into mesenchymal cells [1]. Regulated by microenvironmental signals, this cellular plasticity process is driven by a network of embryonic EMT-inducing transcription factors (EMT-TFs) mainly represented by the SNAIL, TWIST, and ZEB protein families, which interact with epigenetic regulators [2,3]. EMT is not a simple shift from a fully epithelial to a fully mesenchymal state but encompasses a spectrum of multiple states driven by reversible transitions sustaining cell plasticity [4,5]. EMT initiation is a potent source of phenotypic, metabolic, and functional cell plasticity. The expression of EMT-TFs is considered to be transient during embryogenesis, with EMT becoming latent in most adult tissues. However, in addition to fibroblasts, endogenous expression of EMT-TFs has been described in other normal adult differentiated cell types, including melanocytes, that will be the focus of this review, but also endothelial cells [6], neurons [7], and immune cells [8], such as lymphocytes [9], NK cells [10,11], or macrophages [12], suggesting major roles in cell differentiation and normal tissue homeostasis beyond embryonic development.

Aberrant reactivation of this embryonic process in pathological conditions, such as organ fibrosis and tumorigenesis, has been particularly studied in carcinomas [13]. While aberrant expression of EMT-TFs is known to foster dissemination and metastasis [14,15], it also plays a major role in malignant transformation and tumor initiation [16,17,18]. The oncogenic potential of EMT-TFs mainly relies on their ability to promote escape from oncogene-induced senescence and apoptosis by interfering with p53 and RB tumor suppressor pathways [19]. These factors also provide cells with stem-like properties [20,21]. Overall, cells committed to EMT may reside in metastable states, increasing their adaptability to the changing microenvironment that they encounter, both at the primary and distant sites [5].

While a large set of data demonstrated that aberrant reactivation of EMT-TFs in epithelial cells is oncogenic [22], the roles of these factors in non-epithelial cells have not been as thoroughly characterized. Although the term EMT stricto sensu cannot be formally used in the context of non-epithelial-derived cancers, a mesenchymal transition process has been observed in glioblastoma [23] or neuroblastoma [24,25], that are, similarly to melanoma, derived from neural crest cells.

Melanomas display a high degree of inter and intra-tumoral heterogeneity, supported by an exacerbated plasticity of tumor cells. Melanoma intra-tumor heterogeneity does not rely on the classical cancer stem cell (CSC) hypothesis but rather on a “phenotype switching” model, reminiscent of EMT, which is largely dependent on the expression level of the Microphthalmia-associated transcription factor MITF, the master regulator of melanocyte development [26]. According to the rheostat model proposed by Goding [27], high MITF expression maintains differentiated status of melanocytes, intermediate MITF expression sustains proliferation, while low MITF levels generate invasive and slow-proliferating cells with tumor-initiating properties. Melanoma cells were shown to shift in a reversible way between a proliferative and an invasive state [28,29]. Over the years, specific molecular signatures and epigenetic marks discriminating the proliferative and invasive states have progressively been deciphered [30,31]. Aside from MITF, other master transcriptional regulators have been identified, including SOX10 for the proliferative phenotype and AP1/TEAD for the invasive phenotype. We herein review the cell-type-specific roles of EMT-TFs in the melanocyte lineage, melanoma development, phenotype switching, and resistance to treatment.

## 2. Expression and Function of EMT-TFs in the Embryonic Neural Crest and the Melanocyte Lineage

**Figure 1:**
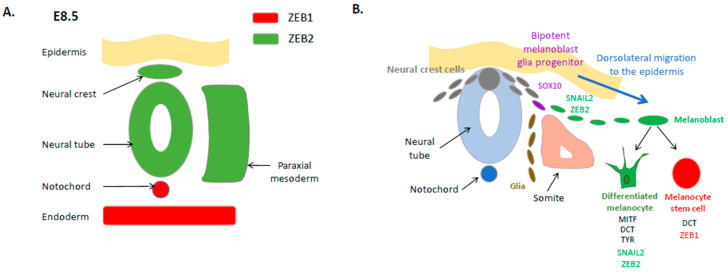
Expression and function of epithelial–mesenchymal transition transcription factors (EMT-TFs) in the embryonic neural crest and the melanocyte lineage. (**A**) Schematic representation of ZEB1 and ZEB2 opposite expression patterns in the mouse embryo at embryonic day E8.5. (**B**) Expression of EMT-TFs during the formation of the melanocyte lineage from the embryonic neural crest. The transcription factor SOX10 is expressed in a bipotent melanoblast/glial progenitor. SNAIL2 and ZEB2 are required for neural crest cell delamination and melanoblast specification and migration through a dorsolateral pathway. SNAIL2 and ZEB2 are also required for the homeostasis of differentiated melanocytes in the epidermis, through the positive regulation of MITF expression. ZEB1 in contrast is expressed in dedifferentiated melanocyte stem cells, which have lost MITF expression. MITF: microphthalmia-associated transcription factor. DCT: dopachrome tautomerase. TYR: tyrosinase.

EMT is essential for many crucial steps during embryonic development, including neural crest cell migration and melanocyte lineage formation. Neural crest cells delaminate from the dorsal neural tube through an EMT process (Figure 1A) enabling them to migrate as individual cells. While individual knock-out of EMT-TF genes can result in significant defects at the gastrulation stage, some redundancy may be observed for neural crest formation. Indeed, mice displaying *Snail1* and *Snail2* mutations still generate neural crests even though they develop multiple craniofacial defects [1]. ZEB2 is also required for neural crest cell development, since its deletion in mice causes embryonic lethality around stage E8.5, with a failure of neural tube closure associated with an early arrest of cranial neural crest cell migration [32]. Tissue-specific knock-out of Zeb2 in early neural crest cell development also results in embryonic lethality, associated with abnormalities in craniofacial, heart, and peripheral nervous system development [33]. In contrast, Zeb1 knock-out in the mouse is not embryonically lethal but induces major defects in skeletal elements and thymic T-cells, leading to perinatal death. Interestingly, expression of ZEB1 and ZEB2 is mainly complementary in mouse embryos, with an overlap in only a limited number of tissues [34]. Indeed, ZEB2 expression around E8.5 is found in the neural plate/crest and the paraxial mesoderm (Figure 1A), while ZEB1 is absent in the neural crest and expressed in the paraxial and limb skeletal elements up to E12, highlighting specific spatiotemporal regulation of the two ZEB family members.

Neural crest cells from the trunk region generate different cells in the developing embryo, namely, melanocytes, neurons, and glia [35]. In the mouse embryo, melanoblasts are specified from a SOX10-positive bipotent melanoblast/glial progenitor (Figure 1B). Upon subsequent gain of expression of MITF, DCT (dopachrome tautomerase) and KIT in melanoblasts, these cells start their dorsolateral migration from E10.5, move into the epidermis around E11 and populate the developing hair follicle. They next separate into two populations: some melanoblasts differentiate into melanocytes and produce the melanin pigment; others dedifferentiate into non-pigmented melanocyte stem cells associated with the loss of MITF and KIT expression. These melanocyte stem cells reside in the hair follicle bulge and replenish hair follicles with new pigmented melanocytes in the subsequent hair cycle.

Melanoblast dorsolateral migration through the developing embryo requires the participation of EMT-TFs, especially SNAIL2 (SLUG) and ZEB2 (SIP1) (Figure 1B). Indeed, *Snai2*-deficient mice display defects in melanoblast specification and migration, resulting in pigmentation abnormalities, including a white forehead [36]. Similarly, in humans, SLUG mutations have been found in some patients displaying skin pigmentation abnormalities, piebaldism, or Waardenburg syndrome type 2 [37,38], a syndrome associated with melanocyte migration defects, which has notably been linked to MITF mutations. ZEB2 is also expressed in migrating melanoblasts in the mouse embryo and their precursor, neural crest cells [39]. Melanocyte-specific (Tyr: Cre) *Zeb2* knock-out induces a drastic hair pigmentation loss in mice that is due to a severe impairment of both melanoblast migration and melanocyte differentiation [39].

In addition to melanoblasts, SNAIL2 and ZEB2 expression is maintained in adult differentiated melanocytes in both mouse and human skin. Indeed, EMT-TF expression analyses in normal human skin samples unexpectedly showed SNAIL2 and ZEB2 expression in differentiated melanocytes, while TWIST1 and ZEB1 were not detected [40]. ZEB2 is required for melanocyte differentiation since ZEB2 knock-down in primary melanocytes induces a down-regulation of MITF and differentiation defects [39]. Overall, these data highlight a major role for SNAIL2 and ZEB2 in the regulation of the homeostasis of the melanocyte lineage, from the developing embryo to the adult life.

Interestingly, while ZEB1 is not expressed in differentiated melanocytes, it may still be involved in the homeostasis of the melanocyte lineage, since it was shown to be expressed in melanocyte stem cells in mouse skin, and could thus be required for the renewal of mature melanocytes from the stem cell pool [39] (Figure 1B). Consistently, in contrast with ZEB2 which activates MITF transcription, ZEB1 was shown to repress MITF expression and alter pigmentation in retinal pigment epithelial cells [41]. More importantly, ZEB2 knock-down in melanocytes not only impairs MITF expression and differentiation, but also results in ZEB1 induction [39]. A switch from ZEB2 to ZEB1 is thus associated with dedifferentiation of melanocytes. Intriguingly, a similar ZEB2/ZEB1 switch has also been described in some immune cell populations. Indeed, while ZEB2 leads to a differentiation of T CD8 lymphocytes towards T effector cells, ZEB1 induces T memory formation [9]. Overall, despite their high degree of similarity at the structure level, the ZEB1 and ZEB2 zinc-finger homeodomain transcription factors display antagonistic expression patterns and functions in the melanocyte lineage.

## 3. EMT-TF Expression Switch during Melanoma Development

**Figure 2:**
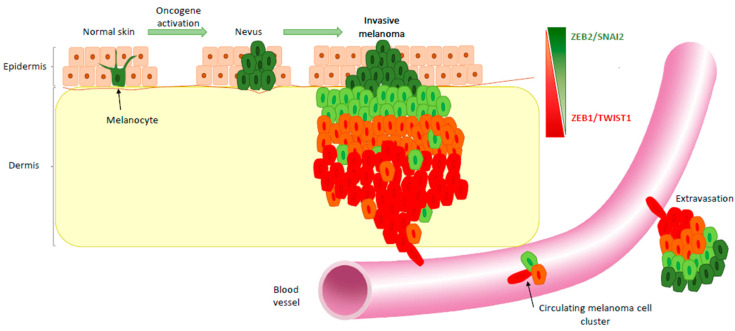
EMT-TF switch during melanoma development. ZEB2 and SNAIL2 are expressed in differentiated melanocytes within the epidermis of normal adult skin. Their expression is maintained in senescent benign nevi. During the conversion to primary melanoma, SNAIL2 and ZEB2 expression is progressively lost in favor of TWIST1 and ZEB1. Moreover, intra-tumoral heterogeneity of EMT-TFs is observed within melanoma lesions, the EMT-TF switch being generally observed as a gradient from upper part to deeper part of invasive melanoma. Gain of invasive capacity upon ZEB1 reactivation promotes melanoma cell entry into the bloodstream, with clusters of circulating melanoma cells, which may display different differentiation expression patterns. The differentiated pattern of EMT-TFs is reproduced in the secondary site following extravasation, allowing metastatic growth.

While EMT-TFs are essential for the development and homeostasis of the melanocyte lineage, they also play major roles during melanoma development. Cutaneous melanomagenesis is a multi-step process initiated by the transformation of a normal melanocyte following an oncogenic insult, leading to primary and metastatic melanoma, passing or not passing through a benign nevus stage (Figure 2). A few years ago, we performed a comprehensive analysis of the expression of the entire EMT-TF network by immunohistochemical staining in human samples representative of melanoma progression [40]. We unveiled a completely different scenario compared to carcinoma. Indeed, we showed that a reversible switch in the expression pattern of EMT-TFs occurs during melanomagenesis. A progressive loss of ZEB2/SNAIL2 and a gain in TWIST1/ZEB1 expression were observed along the transition from melanocytes to malignant melanoma (Figure 2). Moreover, this switch from ZEB2/SNAIL2 to TWIST1/ZEB1 was a significant factor of poor prognosis for melanoma patients. These data are consistent with other independent melanoma series showing loss of ZEB2 [39,42] or SNAIL2 [43]. This ZEB2/ZEB1 switch was also associated with reduced patient survival in an independent cohort [44].

The EMT-TF switch is partly regulated by the BRAF/MEK signaling pathway in normal melanocytes. Inhibition of the MAPK pathway with BRAF/MEK inhibitors induces the opposite reprogramming in human melanoma cells. A double-negative feedback loop involving miR-200 family members and ZEB transcription factors is known to regulate carcinoma cell plasticity [45]. However, miR-200 expression levels are low in melanoma and regulation of ZEB1/2 was shown to be largely miR-200 independent. This reversible switch in EMT-TF expression is rather controlled downstream of ERK by a member of the AP-1 complex, FRA1 (FOS related 1), a master transcription factor of the gene regulatory network of the invasive phenotype in melanoma cells [30]. More importantly, alteration of the switch (through inhibition of ZEB1/TWIST1/FRA1 or ectopic expression of ZEB2/SNAIL2) was sufficient to impair BRAF-dependent melanocyte transformation of melan-a cells both in vitro and in vivo in xenograft experiments.

These results reinforce the notion that ZEB1 and/or TWIST1 not only promote invasive features but also display intrinsic oncogenic functions. Indeed, ZEB1/TWIST1 are expressed in the bulk of primary melanoma and ZEB1 ectopic expression promotes tumorigenic features in melanoma cell lines, while its knock-down drastically decreases the tumorigenic growth of melanoma cells in vivo upon xenograft in nude mice [40,46]. In contrast, ectopic expression of ZEB2 and SNAIL2 in melanoma cells decreases tumor formation in nude mice. Therefore, while ZEB1 and TWIST1 harbor oncogenic activities in melanoma, ZEB2 and SNAIL2, which are expressed in normal melanocytes and are required for their proper differentiation, they may act as oncosuppressive proteins in this specific neural crest-derived lineage. A tumor-suppressive role for SNAIL2 and ZEB2 in melanoma was unexpected, although such an activity was suggested for *Zeb2* mRNA through the activation of PTEN expression [42]. Recent data in transgenic mouse models indicated that *Zeb2* knock-out is not sufficient to promote melanoma initiation in the mouse and even impairs NRASQ61-dependent melanoma formation [44], thus precluding a bona fide tumor-suppressor role. ZEB2-enforced expression in this mouse model also promotes growth of primary and metastatic tumors by favoring melanoma cell proliferation. ZEB2 silencing in mouse NRASQ61 melanoma cells decreases MITF, increases ZEB1, and induces proliferation defects. One can hypothesize that ZEB2 expression follows a model mirroring the MITF rheostat: ectopic expression of ZEB2/MITF in melanoma cells would result in differentiation and cell cycle arrest, while ZEB2/MITF silencing would also result in proliferation defects, an intermediate level of ZEB2/MITF would sustain proliferation of melanoma cells. Accordingly, aberrant expression of ZEB2 in melanoma cells may promote terminal differentiation and decreased tumorigenic capacity upon xenograft in nude mice, while intermediate levels of ZEB2 in transgenic mice would be compatible with proliferation. However, the link between EMT-TFs and proliferation remains unclear since we did not observe any defect in proliferation upon modulation of ZEB1 expression in melanoma cells [40,46].

A reversible switch to a proliferative phenotype may be required for metastasis to develop. Accordingly, the ZEB2/SNAIL2 “differentiation pattern” of EMT-TF expression is replicated in cortical areas of lymph node metastases (Figure 2) similarly to that described in primary tumors, suggesting that melanoma cells may re-differentiate at the metastatic site, reminiscent of the mesenchymal-to-epithelial (MET) process in carcinoma [47,48]. Indeed, reversible EMT-MET cycles are required for metastatic colonization in carcinoma. ZEB2/SNAIL2 might, in this model, equally contribute to the high metastatic propensity of melanomas as previously suggested for SNAIL2 [49] and recently demonstrated for ZEB2 in a mouse model in which ectopic ZEB2 expression facilitates the outgrowth of dormant disseminated melanoma cells and promotes the formation of successful metastases [44].

Alternatively, collective migration of clusters of proliferative and invasive cells may underlie metastatic colonization. Of importance, heterogeneous MITF expression has been detected in circulating melanoma cell clusters [50,51] (Figure 2), consistently with the phenotypic heterogeneity described in breast cancer CTCs (circulating tumor cells), where a partial EMT state sustains their migratory capacity [52]. Monitoring the number and phenotype of CTCs may thus prove highly promising for diagnosis and management of melanoma patients.

Hence, while ZEB1 and ZEB2 are largely co-expressed in mesenchymal tumor cells and display similar oncogenic roles in epithelial cell-derived carcinomas, they display opposite expression patterns and functions in melanocytes and melanoma cells, which partly rely on the antagonistic regulation of the master gene MITF.

## 4. EMT-TFs Drive Phenotype Switching of Melanoma Cells and Intra-Tumor Heterogeneity

The model based on MITF-mediated phenotype switching describes the reversible transitions of melanoma cells [27]. Gene expression analyses of melanoma short-term cultures enabled their classification into two states, proliferative or invasive [53]. Xenograft of either proliferative or invasive cells resulted in tumors displaying a similar level of intra-tumor heterogeneity with MITF^high^ and MITF^low^ cells [28], thus demonstrating that melanoma cells are able to shift in a reversible way between these two states [29]. With regards to EMT-TF expression, cell lines with a MITF^high^ differentiated pattern display a higher level of ZEB2, while invasive/undifferentiated cells present an elevated ZEB1 expression. ZEB1 is one of the top-ranking genes whose expression is inversely correlated with MITF in tumors from the TCGA [30,46]. Moreover, intra-tumor heterogeneity was observed within human primary melanoma lesions by immunohistochemical analyses: opposite gradients in the expression of ZEB2/SNAIL2 and TWIST1/ZEB1 were observed from superficial to deep sites (Figure 2) [40,44]. Intra-tumor heterogeneity in the expression of ZEB2/ZEB1 was also documented in the NRASQ61 melanoma mouse model [44]. Interestingly, analyses of EMT-TF expression in the Jerby-Arnon single-cell data set [54] confirmed that ZEB2 and ZEB1 expressions are anti-correlated. More importantly, a mutually exclusive expression pattern of ZEB1 and MITF was documented in human samples with the presence of MITF^high^/ZEB1^low^ and MITF^low^/ZEB1^high^ subclones [46].

While different studies used ZEB2 and ZEB1 as additional markers to define the proliferative (MITF^high^/AXL^low^) or invasive (MITF^low^/AXL^high^) phenotypes, respectively [55,56], our data further demonstrated that EMT-TFs are major regulators of melanoma cell plasticity that fuel intra-tumor heterogeneity. Indeed, in vitro and in vivo experiments demonstrated that antagonistic functions of EMT-TFs in melanoma at least partly rely on their differing transcriptional regulation of MITF expression. Gene expression profiles of BRAF-activated melanocytes (melan-a cells) revealed that ZEB2/SNAIL2 activate MITF and cause a melanocyte differentiation gene signature, while ZEB1/TWIST1 repress MITF and generate an invasion-associated gene signature [40]. The converse regulation of MITF expression by ZEB1/ZEB2 may rely on differential recruitment of co-activators (p300) or co-repressors (CtBP), similarly to that described for the regulation of TGFβ/BMP target genes [57], though further mechanistic characterizations are needed to confirm this. Gain- or loss-of-function experiments of ZEB1 in human melanoma cells demonstrated that ZEB1 regulates reversible switching between the proliferative and the invasive phenotype [46]. Overexpression of ZEB1 in melanoma short-term cultures is sufficient to drive switching towards a MITF^low^ undifferentiated phenotype bearing stem cell properties, characterized by increased expression of the neural crest stem cell (NCSC) marker NGFR, a major regulator of phenotype switching [46,58]. In contrast, ZEB1 knock-down induces a switch towards a differentiated MITF^high^ phenotype with reduced expression of NGFR. The ectopic expression of ZEB2/SNAIL2 promotes differentiation of melanoma cells, while ZEB2 knock-down results in a reduction of MITF expression and a switch from a differentiated to an undifferentiated melanoma cell phenotype [39]. Accordingly, the switch from ZEB2 to ZEB1 in the NRAS melanoma mouse model is associated with the gain of an invasive phenotype [44]. Similarly, in the BRAF; PTEN melanoma mouse model, when tumors are induced on the tail, which better mimics the melanoma transition from a radial to a vertical growth phase [59], the loss of differentiation markers (such as MITF) in a subset of epidermal melanoma cells, before the dermal invasion, was shown to be associated with ZEB1 activation, reinforcing the role of ZEB1 in this transition. Overall, by regulating MITF-dependent phenotype switching, dysregulation of the EMT-TF network contributes to malignant progression of melanoma.

In addition to FRA-1-driven oncogenic signaling, TGFβ was shown to promote the ZEB2/ZEB1 switch in melanoma cells [44]. The transcription factor GLI2, activated by the TGFβ signaling pathway, was shown to cooperate with ZEB1 to promote *CDH1* repression, thus participating in the mesenchymal transition [60]. Overall, phenotype switching is dictated by both cell-autonomous and non-cell-autonomous mechanisms. The extensive characterization of both intrinsic and extrinsic factors interdependently contributing to EMT processes will be necessary to fully comprehend melanoma phenotype plasticity.

**Figure 3:**
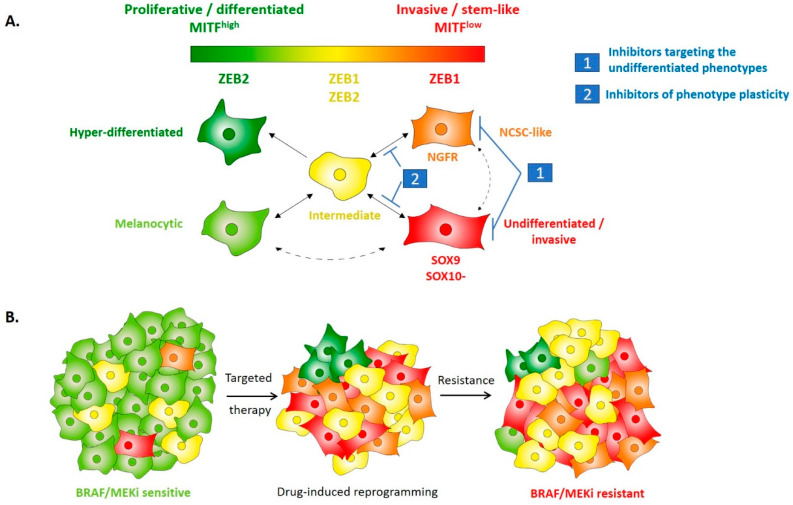
EMT-TFs regulate melanoma phenotype plasticity, intra-tumor heterogeneity and resistance to treatment. (**A**) Schematic model of putative melanoma intra-tumor heterogeneity according to a classification in five cell states, bearing various differentiation capacities, in accordance with their respective expression of MITF. Expected ZEB1 and ZEB2 expressions in the respective phenotypes are indicated. Expression of the main proposed markers of the different cell states is indicated. The invasive and stem-like phenotype actually represents two different states, both expressing high levels of ZEB1. SOX10 expression is present in all but the dedifferentiated state where it would be replaced by SOX9. The intermediate state should co-express ZEB1, ZEB2, and MITF. The intermediate state is believed to be highly metastable and to give rise to the invasive, neural crest stem cell (NCSC) and hyper-differentiated states, especially upon targeted therapy treatment-induced adaptation. Two-way arrows indicate reversible switching between cell states. Dotted arrows indicate putative transitions. The two main therapeutic strategies under investigation are also indicated. (**B**) Evolution of intra-tumor heterogeneity of melanoma during treatment with BRAF/MEK inhibitors-targeted therapy. The ZEB2/MITF melanocytic population is eliminated upon treatment and the tumor size decreases. Only resistant phenotypes remain with an increased proportion of NCSC-like and undifferentiated states. ZEB1-increased expression contributes to drug-induced NCSC reprogramming. Tumor adaptation ultimately leads to resistance with a regain of tumor growth and increased intra-tumor heterogeneity compared to the therapy naive tumor. BRAF/MEK resistant tumors may display high ZEB1 expression in both MITF^low^ and MITF^high^ clones. ZEB1 targeting can induce cell death in BRAFi-resistant melanoma cells, independently of MITF expression level.

With the recent generation of gene expression analyses of tumors at the single-cell level, the phenotype switching model has been refined [54,61,62]. Single-cell RNAseq data of melanoma tumors from Irwin Davidson’s lab [61] described, in addition to the extreme MITF^high^/AXL^low^ and MITF^low^/AXL^high^ states, the existence of a novel intermediate state, with the coexistence of proliferative and invasive features. This intermediate phenotype was shown to co-express both MITF and ZEB1 (Figure 3A). While cell lines preferentially adopt either a proliferative or an invasive phenotype, with a clear-mirrored expression of ZEB1 and MITF, tumors in vivo display a higher level of intra-tumor heterogeneity with a significant proportion of cells in the mixed/intermediate state. This is in accordance with our immunohistochemical staining showing co-expression of ZEB1 and MITF in some clones, especially after development of BRAFi resistance (see below and Figure 3B). Accordingly, in a *SMAD4/7* knock-out mouse model, SMAD4 was required for tumor formation, and a low level of SMAD7 expression promoted an invasive phenotype and metastatic spread associated with ZEB2/ZEB1 switching [63]. Interestingly, in this model, the presence of MITF/ZEB1 double-positive cells was highlighted, with both proliferative and invasive features.

Recent single-cell RNAseq analyses demonstrated that melanoma cells can acquire several intermediate phenotypes. A study performed on mouse patient-derived xenografts (PDXs) by the lab of JC Marine [64], identified four cell states as drug-tolerant when exposed to MAPK inhibitors. These states exhibited transcriptional signatures enriched for neural crest differentiation, response to nutrient starvation, EMT, or pigmentation. This elegant work corroborates the four-state differentiation model proposed by Graeber’s lab [65]. Despite different nomenclatures, these studies pointed to a more complex model of intra-tumor heterogeneity and cell state reprogramming (Figure 3) [66]. Interestingly, ZEB2 expression progressively decreases as the cell state progresses from a differentiated to a more invasive and dedifferentiated phenotype, while a gain in ZEB1 expression is evidenced in the NCSC-like and invasive phenotype.

High levels of ZEB1/TWIST1 expression undoubtedly promote an invasive phenotype in melanoma, including decreased *E-Cadherin*, MITF, and increased expression of *Vimentin*, *SPARC*, and *MMPs* [30,40]. However, ZEB1 also activates NCSC markers (such as NGFR). We thus believe that ZEB1 is able to increase the expression of both NCSC and invasive markers, possibly not in the same cells, or at least not at the same time. This is consistent with models proposed in carcinoma, where ZEB1 may promote stemness features (partial EMT state associated with tumor initiation) but not necessarily invasive/EMT features, these two features being uncoupled [67]. Overall, ZEB2/ZEB1 may not be associated with the acquisition of a given cell state but may regulate reversible transitions of melanoma cell state in a dynamic manner.

## 5. EMT-Like Cell Plasticity as a Driver of Resistance to Treatment in Melanoma

In recent years, melanoma has served as proof-of-concept for the development of both targeted- and immunotherapies and is the archetypal model of tumor resistance [68]. The mechanisms of resistance to BRAF and MEK inhibitors (BRAFi and MEKi) have been extensively described over the years [69]. Concomitantly to the acquisition of genetic mutations, leading to the reactivation of the MAPK pathway, increasing evidence now suggests that non-genetic mechanisms also play a major role in the acquisition of drug resistance. Indeed, approximately 40% of BRAFi/MEKi resistant melanoma cases do not present a known genetic alteration [70]. Resistance has been attributed to drug-induced phenotypic adaptations, including the emergence of invasive or NCSC features, relying on epigenetic, transcriptional, or translational reprogramming [31,71,72,73].

The aberrant activation of an epithelial–mesenchymal transition and the subsequent generation of a cancer stem cell (CSC) phenotype has been largely associated with resistance to therapy in carcinoma, including chemotherapy and targeted therapy such as EGFR inhibitors [74,75]. In melanoma, we provided the first demonstration that ZEB1-mediated phenotype switching is associated with resistance to MAPK inhibitors [46]. Indeed, an elevated level of ZEB1 expression, combined with a low expression of MITF, in melanoma cell lines and patient tumors is associated with an innate resistance to MAPKi. TWIST1 is frequently co-expressed with ZEB1 but is also found in some ZEB1-negative tumors, suggesting that ZEB1 is the main driver of BRAFi resistance, but that TWIST1 may complement ZEB1 when this factor is not activated. ZEB1 and/or TWIST1 expression levels may thus serve as predictive markers of resistance, at least in a proportion of patients (about 30–50%). Furthermore, increased expression of ZEB1 was observed in patient-derived cell lines with an acquired MAPKi resistance and in biopsies from patients experiencing relapse while under treatment (Figure 3B). Gain- or loss-of-function experiments further showed that ZEB1 is sufficient to promote drug-induced reprogramming towards a NCSC state. In accordance with these results, single-cell RNAseq data highlighted the heterogeneous expression of EMT-TFs and also identified an enrichment of cells in a mesenchymal-like phenotype during the treatment of PDXs with BRAF and MEK inhibitors [64,71]. Finally, recent data suggest that both MITF^low^ and MITF^high^ phenotypes may be associated with resistance to targeted therapies [31,66]. Accordingly, we observed that high ZEB1 expression in BRAFi-resistant tumors could be found in both MITF^low^ and MITF^high^ clones [46], and that *ZEB1* knock-down decreased the viability of resistant melanoma cells in both MITF^low^ and MITF^high^ contexts, suggesting that ZEB1 partly functions through MITF-independent mechanisms.

Overall, ZEB1, acting as a transcriptional repressor or activator, owing to its binding to specific co-factors, can down-regulate melanocyte differentiation markers and upregulate melanoma initiating cell markers that cooperate in mediating resistance to MAPKi. Altogether, these studies demonstrate that EMT-TFs are not only markers of specific cell phenotype but define EMT-TFs as part of the central actors controlling melanoma phenotype plasticity and intra-tumor heterogeneity which in turn drive tumor evolution and resistance to current therapies.

In addition to MAPK-targeted therapies, another major breakthrough in the treatment of metastatic melanoma was achieved through immunotherapies targeting negative regulatory checkpoints in immune cells, CTLA4 and PD1 [76]. Nearly 40% of sustained responses were observed with anti-PD1 (Nivolumab, Pembrolizumab), and a combination with anti-CTLA4 may further increase this efficacy [77]. However, despite impressive clinical responses, around 60% of patients still present primary or acquired resistance to these therapies. Mechanisms of resistance to immunotherapy remain unclear. They may rely on tumor microenvironmental properties, including the poor infiltration by immune cells, notably CD8+ T-cells [78]. They likely also involve tumor intrinsic mechanisms, relying on transcriptomic/phenotypic alterations of tumor cells [79]. Indeed, TNFα-induced dedifferentiation of melanoma cells promotes escape from T-cell lysis and resistance to adoptive cell transfer [80]. Activation of the WNT/β-catenin pathway [81,82] or loss of PTEN expression [83] were also shown to promote T-cell exclusion and resistance to immunotherapy in melanoma. Similarly to what was observed with resistance to targeted therapy, preliminary results in a few melanoma patients suggest that immunotherapy may also induce transcriptomic remodeling [84].

The mechanisms by which EMT-dependent cell plasticity mediates immune evasion have been well characterized in carcinoma models, including the regulation of antigen presentation, expression of MHC (major histocompatibility complex), or immune checkpoint ligands such as PD-L1 [85,86,87]. In melanoma, evidence also suggests that EMT-TFs may contribute to immune escape, as illustrated by the role of SNAIL in the recruitment of Treg lymphocytes [88]. Data obtained in transplanted mouse models of carcinomas also suggested that mesenchymal tumors may display increased resistance to immunotherapy compared to epithelial tumors [87]. However, the role of EMT in the resistance to immunotherapy needs to be further investigated in human tumors. Indeed, the “IPRES” (innate PD1 resistance) signature described by the team of Ribas [89] in melanoma, revealed an enrichment in a “MAPK inhibitor-induced EMT” mesenchymal signature, which includes AXL, WNT5A, or TWIST2, for example, but neither the ZEB nor the SNAIL family members. However, bulk RNAseq analyses have limitations and may be biased since EMT-TFs are expressed in many cell types in the tumor microenvironment, including cancer-associated fibroblasts (CAFs), endothelial cells, but also immune cells (T cells, macrophages, and NK cells). In this respect, inactivation of endothelial ZEB1 expression was recently shown to sensitize tumors to anti-PD1 [6]. Overall, changes in EMT-TF expression levels in tumor cells may be hidden by modulations within the tumor microenvironment, thus emphasizing the requirement for scRNAseq analyses and/or the development of in situ analyses of tumors to better tackle the issue of intra-tumor heterogeneity.

## 6. Perspectives: Targeting of Cell Plasticity to Prevent Resistance to Treatment

Despite recent progress in the treatment of metastatic melanoma, the emergence of resistance and/or toxicities to both targeted- and immunotherapies remains a major barrier to complete remission. A better understanding of cellular and molecular mechanisms underlying phenotypic adaptations, and thus the exceptional capacity of melanoma cells to develop resistance to current therapeutic strategies, may help to define biomarkers of response and new combination therapies.

We have already obtained proof-of-concept that ZEB1-knock-down re-sensitizes resistant cells to BRAF/MEK therapy [46], consistent with data in carcinoma [90]. Therefore, targeting the EMT-TF network represents an attractive treatment strategy for metastatic melanomas. However, the targeting of EMT as a highly reversible plasticity process is a challenging issue, given that EMT and MET cycles are required at different stages of tumor progression. Indeed, reverting EMT could result in the outgrowth of tumor cells already seeded in a metastatic niche. Strategies aiming at preventing the mesenchymal transition at an early stage, thus preventing dissemination, or at targeting the dedifferentiated states are still under investigation, including targeting of the TGFβ pathway [91] (Table 1 and Figure 3).

Accordingly, a promising approach was based on methotrexate-mediated upregulation of MITF, which results in the differentiation of melanoma cells, an abrogation of their migratory capacities, and sensitization to the cytotoxic agent TMECG [99]. Other therapeutic strategies aim at combining conventional therapies with drugs specifically targeting undifferentiated/invasive melanoma cells. Targeting of AXL in the invasive state, thanks to an AXL antibody linked to a microtubule-disrupting agent, cooperates with MAPK inhibitors in inhibiting tumor growth [92]. Similarly, inhibition of the retinoic receptor RXR, a key driver of the NCSC identity, synergizes with MAPK inhibitors in vitro and increases the tumor latency following xenograft [64]. Interestingly, undifferentiated melanoma cells were also shown to display an altered metabolic activity with a reduced glutathione level compared to differentiated cells [65]. The diminished glutathione quantity is correlated with an increased sensitivity to ferroptosis induced by Erastin treatment. Erastin effectively synergizes with BRAF inhibitors and decreases the number of persistent dedifferentiated melanoma cells in vitro. Accordingly, drugs affecting cell metabolism and more specifically inducing ferroptosis have also shown promising results in other cancer models with a mesenchymal phenotype [98]. Since metabolic reprogramming is intimately linked to EMT, promising pharmacological inhibitors of metabolic pathways are currently investigated for their capacity to target EMT [96,97].

Finally, epigenetic plasticity has been proposed to account for the rapid adaptation of melanoma cells to treatment. Multiple epigenetic modifiers have been shown to regulate EMT-TF expression or to directly interact with EMT-TFs [100]. It would be of utmost interest to define the network of epigenetic regulators that cooperate with EMT-TFs in melanoma to drive epigenome remodeling and rapid phenotype switching of melanoma cells in order to identify relevant epidrugs. Notably, inhibition of the histone methyltransferase EZH2 has been shown to promote dedifferentiation of melanoma cells, restore immunogenicity, and re-sensitize tumors to immunotherapy [101]. HDAC inhibition has also been reported to increase response to immunotherapy [93,94]. Several clinical trials investigating the combination of epigenetic drugs with MAPK inhibitors or immunotherapy are ongoing and the results will reveal the feasibility of such therapeutic approaches in melanoma [95,102].

## 7. Conclusions

In conclusion, recent evidence has started to unveil the specific expression patterns and functions of EMT-TFs in the melanocyte lineage and melanoma, compared to epithelial-derived cancers. Overall, EMT-TFs appear as major regulators of phenotype switching in melanoma, between differentiated/proliferative and neural crest stem cell-like/invasive states, contributing to the emergence of resistance to current therapeutic strategies in melanoma. Further research is still needed to characterize the precise molecular mechanisms by which EMT-TFs mediate these reversible transitions, taking into account the plastic intermediate states. Single-cell approaches and in situ analyses of melanoma patient samples, upon treatment, will be of major interest in order to more precisely address EMT-TFs intra-tumor heterogeneity and their crosstalk with the immune microenvironment. Finally, future investigations will decipher the molecular crosstalk of EMT-TFs with epigenetic and metabolic regulators. This should lead to the development of novel therapeutic strategies aiming at targeting cell plasticity, which may be tested in combination with targeted- or immunotherapies.

## Figures and Tables

**Table 1:** Short list of the main therapeutic strategies under evaluation to target EMT-dependent cell plasticity in cancers.

Strategy	References	Target	Drug/Compound	Clinical Trial	Trial ID	Cancer Type
TGFβ pathway inhibitors	[91]	TGFβ monoclonal antibody	GC1008 (fresolimumab)	Phase I	NCT00356460	Renal Cell Carcinoma and Malignant Melanoma
AXL targeting	[92]	AXL small molecule inhibitor	BGB324	Phase I/II in combination with either dabrafenib/trametinib or pembrolizumab	NCT02872259	Metastatic Melanoma
Epigenetic drugs	[93,94]	HDAC inhibitors	Entinostat	Phase II in combination with erlotinib	NCT00602030	Non–Small-Cell Lung Cancer
	[95]		Vorinostat	Phase I	NCT02836548	Resistant BRAFV600 melanoma
Metabolic inhibitors	[96,97]	Inducible nitric oxide synthase (iNOS)	L-NMMA	Phase Ib/2 in combination with Taxane	NCT02834403	Triple Negative Breast Cancer
Ferroptosis inductors	[65,98]	GPX4 (through gluthatione depletion)	PRLX 93936 (Erastin analog)	Phase I	NCT00528047	Advanced Solid Tumors
